# Human immune system adaptations to simulated microgravity revealed by single-cell mass cytometry

**DOI:** 10.1038/s41598-021-90458-2

**Published:** 2021-06-07

**Authors:** J. M. Spatz, M. Hughes Fulford, A. Tsai, D. Gaudilliere, J. Hedou, E. Ganio, M. Angst, N. Aghaeepour, Brice Gaudilliere

**Affiliations:** 1 Department of Medicine, Metabolism Division, San Francisco Department of Veterans Affairs Medical Center, San Francisco, CA USA; 2 Department of Medicine and Department of Surgery, University of California, San Francisco, CA USA; 3 Department of Anesthesiology, Perioperative, and Pain Medicine, Stanford University School of Medicine, 300 Pasteur Dr. Rm S238, Grant Bldg, Stanford, CA 94305 USA; 4 Department of Surgery, Plastic Surgery Division, Stanford University School of Medicine, Stanford, CA 94305 USA; 5 Department of Biomedical Data Sciences, Stanford University School of Medicine, Stanford, CA USA; 6 Department of Pediatrics, Stanford University School of Medicine, Stanford, CA USA

**Keywords:** Experimental models of disease, Aerospace engineering, Immunology

## Abstract

Exposure to microgravity (µG) during space flights produces a state of immunosuppression, leading to increased viral shedding, which could interfere with long term missions. However, the cellular mechanisms that underlie the immunosuppressive effects of µG are ill-defined. A deep understanding of human immune adaptations to µG is a necessary first step to design data-driven interventions aimed at preserving astronauts’ immune defense during short- and long-term spaceflights. We employed a high-dimensional mass cytometry approach to characterize over 250 cell-specific functional responses in 18 innate and adaptive immune cell subsets exposed to 1G or simulated (s)µG using the Rotating Wall Vessel. A statistically stringent elastic net method produced a multivariate model that accurately stratified immune responses observed in 1G and sµG (*p* value 2E−4, cross-validation). Aspects of our analysis resonated with prior knowledge of human immune adaptations to µG, including the dampening of Natural Killer, CD4^+^ and CD8^+^ T cell responses. Remarkably, we found that sµG enhanced STAT5 signaling responses of immunosuppressive T_regs_. Our results suggest µG exerts a dual effect on the human immune system, simultaneously dampening cytotoxic responses while enhancing T_reg_ function. Our study provides a single-cell readout of sµG-induced immune dysfunctions and an analytical framework for future studies of human immune adaptations to human long-term spaceflights.

## Introduction

During spaceflight, suppression of the immune system is a well-documented consequence of both short- and long-duration missions^1–9^. The dysregulation of immunological mechanisms have been studied in ground simulations of microgravity (µG)^8–11^, in human immune cells^4,12,13^ and in mice (astromice)^8,9^ during spaceflight, and in astronauts^9,12,14,15^. Transcriptomic analyses of human peripheral blood mononuclear cells (PBMCs), splenocytes and purified T cells activated in µG during spaceflight have demonstrated that the absence of gravity profoundly inhibits the capacity of immune cells to respond to in-vivo^8^ and ex-vivo stimulations^8,9,12,14,15^. Evidence of enhanced virulence of pathogens^16,17^ and increased viral shedding in astronauts exposed to µG presents potential mission-critical risks for long-duration, deep space exploration^18–20^.

Emerging high-content, immune-profiling technologies, including mass cytometry, provide powerful means for the single-cell evaluation of complex physiological immune responses, such as that produced by µG^9,11–15,21–24^. Here, we utilized a 41-parameter mass cytometry approach to comprehensively profile the effect of µG on human PBMC surface activation markers and intracellular signaling responses, cultured in the NASA developed Rotating Wall Vessel, one of the most commonly used models of simulated (s)µG^25,26^. Other established sµG models include the two-dimensional clinorotation and the Random Positioning Machine models^9,27–31^. The primary goal of the study was to expand current knowledge anchored in bulk transcriptomic profiles of PBMCs^4,12,14,15^ by adding a single-cell and proteomic readout of major immune cell effector function after exposure to sµG. Ultimately, this research will identify modifiable targets that can be exploited to decrease µG-induced immunological dysregulation.

## Results

### Transcriptomic assessment of peripheral immune cell adaptations to sµG recapitulates spaceflight observations

**Figure 1:**
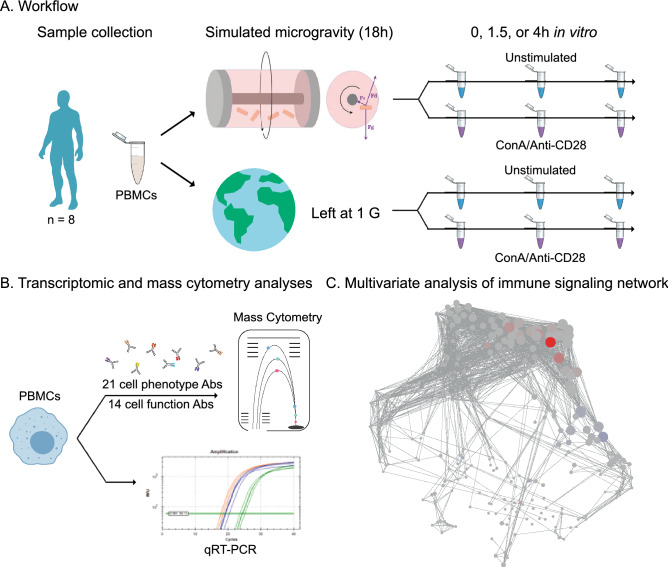
Experimental workflow. (**A**) Blood samples from eight healthy volunteers were collected. Peripheral blood mononuclear cells (PBMCs) were isolated and exposed to simulated microgravity (sµG) or (1G) (static control) using a random positioning machine for 18 h, then stimulated with ConA and anti-CD28 (ConA/anti-CD28) or left unstimulated. (**B**) The activation and intracellular signaling responses of all major immune cell subsets were quantified using single-cell mass cytometry. In parallel, the mRNA expression of a select number of genes was quantified using qRT-PCR. (**C**) The high-dimensional immunological dataset was visualized as an immune signaling correlation network. Cell-type-specific immune responses that differed between the sµG and 1G conditions were identified using a multivariate Elastic Net (EN) method.

PBMC samples from eight healthy adult donors were purchased (Stanford Blood Center, Stanford, CA) for this study. PBMCs were loaded into rotating-wall vessels and exposed to 18 h of either 1-gravity (1G, static control) or sµG. The 18 h sµG exposure was chosen based on prior studies examining the kinetics of transcriptional changes in T-cells in response to sµG showing profound effect on T cell mRNA and microRNA expression after 18 h exposure^9,13^. We reasoned that the 18-h would allow simultaneous detection of mRNA changes and post-translational protein modifications (e.g. phosphorylation of kinases and transcription factors) that temporally coincide with sµG-induced changes observed at the mRNA level. PBMCs were analyzed at baseline (0 h, unstimulated) and following a 1.5 h or 4 h activation with a combination of Concanavalin A and anti-CD28 (ConA/Anti-CD28). Stimulation with ConA/Anti-CD28 provides a robust means to activate multiple signaling responses critically implicated in the proliferation, survival, and differentiation of innate and adaptive cells^32,33^ was used to conform to previous studies of human PBMCs after exposure to µG (both spaceflight and sµG). For each gravity condition, two cell aliquots were obtained and subjected in parallel to (1) pooled and bead isolated Th cell gene arrays and transcriptomic analysis (qRT-PCR) of select genes previously shown to be altered by sµG and spaceflight in immune cells (including interleukin 2 receptor [IL2R]α, tumor necrosis factor [TNF]α, CD69, CLL4 and Interferon [IFN]γ^4,12,14,15^); or (2) single-cell proteomic analysis with mass cytometry (Fig. 1).

**Figure 2:**
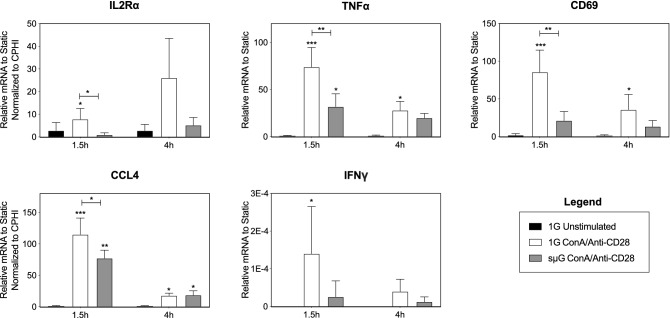
sµG alters immune cell transcriptional responses to ConA/anti-CD28 stimulation. Differential expression of indicated mRNA transcripts isolated from PBMCs exposed to sµG or 1G for 18 h then stimulated with ConA/anti-CD28 for 1.5 h or 4 h. Bar plots show median with standard deviation (n = 8). *indicates *p* < 0.05, **indicates *p* < 0.01, ***indicates *p* < 0.001.

Results from the qRT-PCR analysis revealed no significant differences in baseline (unstimulated) mRNA expression after 18 h of 1G or sµG exposure. In contrast, in PBMCs stimulated with ConA/Anti-CD28, exposure to sµG decreased the mRNA expression of the interleukin 2 receptor (IL2R)α, (this subunit is required for full T cell activation), tumor necrosis factor (TNF)α, CD69, and CLL4 at the 1.5 h or 4 h stimulation time points. A trend towards decreased interferon (IFN)γ mRNA response after sµG exposure was also observed (Fig. 2). Our data indicate that exposure to sµG results in the broad inhibition of immune cell capacity to respond to a potent activating stimulus (ConA/Anti-CD28) and are consistent with previous transcriptomic analyses of human immune cells exposed to the spaceflight environment^9,12–15^. To determine whether differential mRNA expression translated into functional and cell-specific differences in immune cell signaling behavior, we employed a high-dimensional, single-cell mass cytometry approach.

### Exposure to sµG produces system-wide and cell-specific alterations of immune cell responses

PBMCs exposed to 1G or sµG were analyzed using a 41-parameter mass cytometry immunoassay. The approach allowed for the simultaneous assessment of 252 functional responses measured in 18 innate and adaptive immune cell subsets that were manually gated according to established flow cytometry guidelines (Fig. S1)^34^. Before investigating functional responses, we first determined the effect of sµG on the relative abundance of individual cell subsets. The analysis showed no significant difference in immune cell abundance between the 1G and sµG conditions (False Discovery Rate, FDR, q > 0.01, Fig. S2). This was expected since the short-term incubation time used in our experiment is not enough time to detect meaningful differences in cell proliferation.

**Figure 3:**
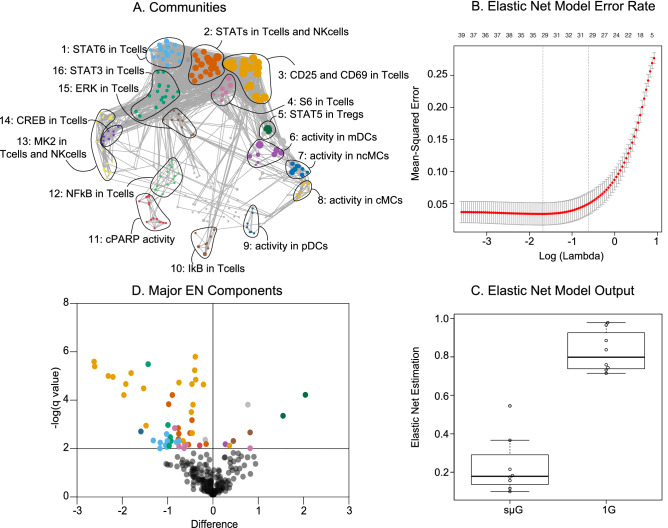
Multivariate modeling of immune cell adaptations to sµG analyzed using mass cytometry. (**A**) Correlation network depicting single-cell immune responses to ConA/anti-CD28 stimulation after sµG or 1G exposure. Nodes represent the protein expression or phosphorylation level of one of 14 functional proteomic markers for a given immune cell subset (asinh ratio relative to the unstimulated condition). Edges are proportional to the Spearman correlation between two nodes. The network segregates into 16 communities that were characterized based on the functional attribute that appeared most frequently in each community. (**B**) An EN method analysis of immune cell responses that differentiated samples exposed to sµG or 1G. The graph depicts model error rates after cross-validation across a range of regularization threshold [log(lambda)]. EN models with the lowest error rate (left vertical dotted line) and one standard deviation from the lowest error rate (right vertical line) are indicated. (**C**) EN model output (*p* = 2E−4, Wilcox signed rank test, n = 8). (**D**) The dot plot illustrates -log (q value) comparing the sµG and 1G conditions (after 4 h ConA/anti-CD28 stimulation). Horizontal line at y = 2 signifies a significance level of 0.01. Significant features above the grey line are colored according to their respective community. Discovery determined using the Two-stage linear step-up procedure of Benjamini, Krieger and Yekutieli, with q = 0.01.

To evaluate the effect of sµG on immune cell function, 14 functional responses—including the phosphorylation of canonical elements of the JAK/STAT, MAPK, and NF-κB signaling pathways and the expression of cell activation markers such as IL2R (CD25) and CD69 were assessed in each immune cell subset as the difference (asinh ratio) between the unstimulated and the 4 h stimulation conditions (Fig. 3). The resulting high-dimensional mass cytometry dataset yielded a correlated network that emphasized the connectivity of immune cell functional adaptations after sµG exposure. The correlation network divided into 16 major communities of highly correlated immune responses, which were annotated based on the signaling property, surface marker expression, and/or cell subset most highly represented (Fig. 3A).

A multivariate Elastic Net (EN) method—a regularized regression method, which performs particularly well for the analysis of high-dimensional, inter-correlated data^11,35,36^ was utilized to determine whether immune cell responses differed after 1G and sµG exposure. The EN algorithm identified a robust (*p* value = 2E−4, Fig. 3B,C) multivariate model that stratified immune cell responses after 1G and sµG exposure. Statistical significance of the EN model was established using a stringent cross-validation method (Fig. 3B). These results, derived from the single-cell and proteomic analysis of immune cell responses, corroborate our transcriptomic data and indicate that exposure to simulated sµG markedly alters the functional organization of the human immune signaling network.

### ***sµG suppresses CD4***^+^***, CD8***^+^***, and NK cell effector responses while enhancing T***_***reg***_*** responses***

The EN approach produced a multivariate signature of cellular adaptations after exposure to sµG. Examination of individual EN model components plotted according to increasing FDR and effect size facilitated the biological interpretation of the multivariate EN output (Fig. 3D, Table S2). The most informative components appeared within three communities characterized by the functional attributes CD25 (10 EN components, community 3), CD69 (11 components, community 3), or elements of the JAK/STAT signaling pathways (26 EN components, communities 2 and 5) respectively.

**Figure 4:**
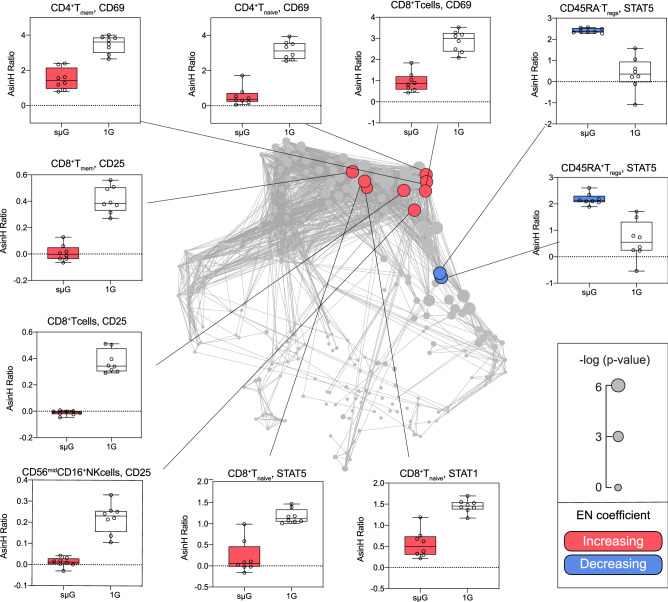
EN Model components reveal cell-specific immunosuppressive effect of sµG on innate and adaptive immune cell subsets. *Center.* EN model components are visualized on the correlation network. Blue/red color scheme indicates increased/decreased EN component (respectively) in the sµG vs. the 1G condition. Sizes of nodes correspond to -log (*p* value) (Wilcox signed rank test, n = 8). *Periphery.* Box plots depicting major EN model components (AsinH Ratio of the stimulated samples compared to unstimulated samples). Additional model components are listed in Table S2.

EN components pointed at immune cell adaptations to sµG that were consistent with our transcriptomic findings (Fig. 4). For instance, sµG exposure dampened the IL-2R (CD25) protein expression in response to ConA/Anti-CD28 in multiple innate and adaptive immune cell subsets, including CD56^dim^CD16^+^NK cells (q = 2.2E−5), and CD8^+^T cell subsets (q = 2.0E−6). Similarly, sµG inhibited CD69 responses to ConA/Anti-CD28 in CD4^+^ and CD8^+^ T cells in both naïve and memory compartments (q ranging from 3E−6 to 7.2E−3). SµG also inhibited JAK/STAT signaling (primarily pSTAT1 and pSTAT5) responses in CD8^+^T cell subsets compared to 1G (q ranging from 6.0E−5 to 4.1E−3), an effect that is consistent with observed transcriptional inhibition of IFNγ, which activates JAK/STAT1 and signaling responses^37^. These results dovetail with prior transcriptomic and flow cytometry studies and suggest that sµG inhibits important immune cell responses implicated in defensive immunity, notably against viral pathogens, such as CD8^+^T and NK cytotoxic responses^38^.

The analysis also revealed surprising and cell-type specific effects of sµG, particularly within the regulatory T cell (FoxP3^+^T_regs_) compartment (Fig. 4). In contrast to inhibiting the pSTAT5 responses in CD8^+^T and NK cells, sµG increased the pSTAT5 response in T_regs_ (both naïve and memory T_regs_) compared to 1G despite the reduction of CD69 in microgravity. The frequency of T_regs_ was not affected by sµG, as expected since the time point was only 4 h, implying that a change in T_reg_ numbers did not contribute to observed STAT5 differences. STAT5 activity is critical for the differentiation, stability, and immunosuppressive function of peripheral T_regs_^39,40^. The differential effect of sµG on STAT5 signaling observed for T_regs_ and CD8^+^ T cells suggests that several immunosuppressive mechanisms synergize after exposure to sµG: sµG simultaneously dampens CD4^+^ T, CD8^+^ T and NK cell capacity to respond to a robust activation stimulus (decreased CD25, CD69, and JAK/STAT responses) while enhancing immunosuppressive T_reg_ responses (increased STAT5 responses).

Observed sµG effect on peripheral immune responses to ConA/anti-CD28 could be the result of differences in immune response capacity to stimulation or, alternatively, to differences in basal signaling activity. To determine whether sµG alters basal immune cell signaling activities, we performed an EN analysis comparing samples exposed to 1G or sµG (18 h) in the absence of ConA/anti-CD28. The analysis identified a cross-validated EN model differentiating the 1G and sµG (*p* value = 3.7E−4, Fig. S3). Examination of the most informative features of the basal EN model showed that the basal STAT5 signaling activity in Treg subsets and basal MyD88 signaling activity (including increased MAPKAPK2, P38, NFkB and EKR1/2 signaling activities) in cDCs were lower after exposure to sµG than to 1G (Table S3). In contrast, basal signaling responses in CD4^+^T cell (other than Tregs) or CD8^+^T cell subsets did not contribute to the EN model significantly. The results suggest that alteration in basal immune cell signaling tone contributes, at least partially, to observed differences in immune responses to ConA/anti-CD28.

## Discussion

Immunosuppression during spaceflight has been recognized since the Apollo missions and remains a major health risk for astronauts, particularly in the development of opportunistic viral infections^10,18–20,41^. This study provides an in-depth and functional assessment of the effect of sμG on the human peripheral immune system. Analysis of the high-dimensional mass cytometry dataset produced by this study identified profound and cell-specific immune alterations caused by sμG that spanned multiple innate and adaptive cell compartments. Notably, sμG suppressed key aspects of CD4^+^, NK cell, and CD8^+^ T cell activation (including CD25, CD69, and JAK/STAT signaling) while enhancing STAT5 signaling responses in T_reg_ cells.

High parameter technologies such as mass cytometry have transformed our ability to functionally assess the human immune system in response to extreme physiological stressors. Previous studies focused on analyses of circulating inflammatory cytokines, transcriptomic assessment of bulk or isolated immune cells, or the functional evaluation of select immune cell subsets have provided important insight on the immunosuppressive effect of sμG. However, the lack of single-cell resolution or the limited number of proteomic parameters precluded a comprehensive and functional assessment of all major immune cell subsets. In this study, application of mass cytometry combined with a machine learning approach provided a statistically stringent multivariate model characterizing the effect of sμG on over 250 individual immune cell functional attributes. Demonstration of the utility of high-dimensional immune profiling and adapted analytical approaches to study μG in ground-based experiments provides the foundation for future analyses of human immune adaptations during short- and long-term spaceflights.

The multivariate analysis pointed at immune cell alterations that were, for the most part, in agreement with prior analyses of μG’s immune modulation^6–9,12,14,42,43^. For example, B cell responses were largely unchanged after sµG exposure. This result is in agreement with prior studies showing that B cell homeostasis is preserved during long-term spaceflight^44^. Consistent with prior analyses of NK cell function during long-term spaceflight^42^, our data suggest that sμG profoundly inhibits CD25 and CD69 expression in NK cell subsets, which are important markers of proliferative and cytotoxic capacity for these cell subsets. Similarly, sμG impaired multiple aspects of CD8^+^ T cell function, including CD25, CD69, and JAK/STAT1 and STAT5 signaling responses. The observed suppression of NK and CD8^+^ T cell function by sμG dovetails with prior documentation of clinically significant impairment of viral pathogen defenses during short and long-term spaceflights, including the re-emergence of latent viruses, such as herpes simplex virus (HSV-1), Epstein-Barr virus (EBV), cytomegalovirus (CMV) and varicella zoster virus (VZV)^18–20,41^.

The single-cell resolution afforded by mass cytometry enabled novel observations. Remarkably, one of the most significant differences observed was an increased STAT5 signaling response in T_regs_ with sμG exposure. Additional analyses of the basal mass cytometry dataset suggested that observed effect of sμG on STAT5 signaling response in T_regs_ resulted from alterations of basal T_reg_ signaling activity as well as T_reg_ signaling response capacity to stimulation with ConA/anti-CD28. These results highlight one of the advantages inherent to the use of a high-parameter immunoassay, as this finding would have likely been undetected in the absence of simultaneous phenotypic and functional assessment of individual CD4^+^ T cell subsets. Activation of the transcription factor STAT5 downstream of IL-2 is a critical signaling event for the differentiation and suppressive function of peripheral T_regs_^40,45^. Our data suggest that enhancement of T_reg_ suppressive capacity via increased STAT5 signaling activity is a plausible mechanism contributing to astronauts’ immunosuppression observed in μG^38^. Interestingly, there was no difference in CD25 expression in T_regs_ between the sμG and 1G conditions, suggesting that sμG-induced STAT5 signaling activation does not require upregulation of the IL-2R. Rather, CD25-independent mechanisms may be implicated in the sμG-regulation of STAT5 signaling in T_regs_, such as activation of Protein Phosphatase 2A, which was recently shown to enhance STAT5 signaling response in T_regs_ in the absence of CD25 upregulation^46^. In contrast, CD69 expression was decreased in all CD4^+^ T cell subsets, including T_reg_ cells. These results emphasize the complexity of sμG immune modulation and suggests that while sμG enhances STAT5 signaling responses in T_regs_, other aspects of T_reg_ suppressor functionality are impaired. The latter observation is consistent with prior in vivo adoptive transfer studies in mice on a 15-day spaceflight (STS-131), showing impairment of immune tolerance to ovalbumin in mice exposed to μG compared to 1G^8^.

The study has certain limitations. The use of sμG as a model system limits the generalizability of the findings. Unarguably, spaceflight is the ideal environment for studying immune cell function in μG. However, sμG experiments using validated models, such as the RWV, the two-dimensional clinorotation or the RPM models^9,27–31^, are an important experimental paradigm for the cost-effective discovery of novel biology that can then be tested in confirmatory in-flight experiments. Importantly, we have compared previous data from spaceflight and sμG of the rotating wall vessel and random positioning machine and have found that expression of measured transcripts (including IL2Rα, TNFα, CD69, and CCL4) was consistent between different sμG models and between sμG and spaceflight^9,12,14,39^. In addition, our study design focused on a single sμG exposure time point. Future experiments examining additional time-points of T-cell changes to microgravity are warranted and should be high-priority scientific objectives for a future spaceflight mission. While the mass cytometry immunoassay allowed measurement of over 40 parameters per immune cell, the list of phenotypic and functional markers is not exhaustive. Similarly, immune cells were evaluated in response to a single stimulation condition, which limited analysis of their functional response. However, our approach provides the analytical basis for future work aiming at an exhaustive characterization of μG-mediated immune modulation.

In summary, we employed high-parameter mass cytometry to produce a single-cell, functional atlas detailing the effects of sμG on the human immune system. Our findings indicate that sμG dampens important innate and adaptive immune cell effector functions while increasing suppressive immune cell function. μG thus orchestrates a multi-cellular immunosuppressive response that may contribute to impairment of pathogen defense. Our study in human immune cells exposed to sμG provides the experimental and analytical framework for future spaceflight studies that will allow the data-driven development of interventions designed to mitigate the clinical consequences of immunosuppression during spaceflight.

## Materials and methods

All procedures performed in this study involving human blood samples were in accordance with the ethical standards of the Institutional Review Board of Stanford University and with the 1964 Helsinki Declaration and its later amendments or comparable ethical standards. De-identified peripheral whole blood samples were obtained from eight healthy human donors between the ages of 21 and 55 years from the Stanford University Blood Center. PBMCs were isolated using a Ficoll gradient method. PBMCs were counted and re-suspended in 31 ml of complete media at 3E6 cells/ml (RPMI, 10% Fetal Bovine Serum, 1% L-Glutamine, 1% Penicillin). At experimental start time (T = 0 timepoint) 10 ml of the cell suspension was loaded into three 10-ml disposable rotating (15 rpm) wall per timepoint (Synthecon, Houston, TX): (1) 1G static control, vehicle treatment, activation at experimental time point 18 h for (2) 1G ConA/Anti-CD2 (3) sμG ConA/Anti-CD28. At indicated timepoints (Fig. 1), 1.5 ml (4.5E6) and 500 μl (1.5E6) of cell suspension were removed and volume replaced with complete media from each rotating-wall vessel for gene expression and mass cytometry respectively. Suspended cells are rotated synchronously in the vessel such that the fluid dynamic effect on them mimics a particle allowed to free fall in a column of fluid. The time-averaged gravitational vector on individual cells is a residual 10^−3^ g force that approximates μg^9,25^, cell suspensions used for RNA isolation were spun down at 300-g and suspended in 1 ml of RNACellProtect Reagent (Qiagen, Valencia, CA, USA) for downstream RNA processing and qRT-PCR. An equal volume of complete media was immediately added to the cell suspension for mass cytometry analysis and fixed using 100 µl of 16% paraformaldehyde (1.5% final concentration) for 10 min at room temperature. Samples were washed twice with phosphate-buffered saline and volume was adjusted to 100 µl for downstream mass cytometry analysis.

### qRT-PCR analysis

Total RNA extraction was performed following standardized, previously published techniques using RNAEasy (Qiagen, Valencia, CA, USA) protocols^13^. Details of qRT-PCR methods have been previously published^13^. At the end of the amplification period, melting curve analysis was performed to confirm the specificity of the amplicon. RNA samples were normalized to cyclophilin A (CPHI), also known as kinase C peptidyl prolyl isomerase A (PPIA), as an internal standard. PPIA expression is stable between normal gravity and μG conditions. Relative quantification of gene expression was calculated by the 2 − ΔΔ*Ct* equation. All data derived using qRT-PCR were from independent donor biologic samples.

All data were checked for normality, and standard descriptive statistics computed. Overall treatment and sμG effects were evaluated using analysis of variance (2-way ANOVA) for all continuous variables. Fisher’s exact test was used to determine whether differences between groups were significant. Differences were considered significant at *p* < 0.05. Data are reported as mean ± SD, unless otherwise noted.

### Sample barcoding for mass cytometry analysis

To minimize the effect of experimental variability on mass cytometry measurements between samples from different treatments, samples were barcoded, as previously described, using unique combinations of three out of six palladium (Pd) isotopes to enable the simultaneous staining and analysis of 20 different samples^47^. This barcoding strategy minimized the impact of experimental variability on the EN model’s false-positive rate as the EN was built against the two gravity conditions.

### Antibody staining and mass cytometry analysis

Antibody staining and mass cytometry analyses were performed according to established guidelines^48^. Antibody staining and mass cytometry analyses were performed using metal-conjugated antibodies against 21 surface and 14 intracellular markers which were chosen to characterize major immune cell types for functional analysis of signaling responses and activation marker expression. Antibodies were obtained either pre-conjugated from the manufacturer (Fluidigm, South San Francisco, California) or were conjugated by the investigators with the appropriate metal isotopes. Purified unconjugated antibodies in protein-free PBS carrier were labeled using the MaxPAR antibody conjugation kit (Fluidigm) following the manufacturer’s instructions. All antibodies used in the analysis were titrated and validated on samples that were processed identically to the samples in the study. Antibodies were used at concentrations listed in Table S1. Barcoded and antibody-stained cells were analyzed on a Helios mass cytometer (Fluidigm, Inc.).

### Derivation of immune features

Manual gating of immune cells from mass cytometry data identified classical and non-classical monocytes (cMC and ncMC respectively), NK cells, B cells, myeloid dendritic cells (mDCs), CD4^+^ and CD8^+^ T cells (naïve and memory T cells respectively), T_regs_, *γδ*T cells, and their subsets for a total of 252 unique immune-cell subsets (Fig. S1)^34^. For each cell subset, the mean signal intensity of functional markers was quantified, including phosphorylated (p)STAT1, pSTAT3, pSTAT5, pNF, pNFκB, total IκB, pMAPKAPK2, pP38, prpS6, pERK1/2, and pCREB as well as CD69 and CD25. Intracellular signaling response features were derived from analysis of signal intensity of all functional markers in samples stimulated with ConA/Anti-CD28. Functional responses in each cell type were calculated as the difference in median signal intensity (asinh ratio) of each signaling protein between the 0 h (unstimulated) and 4 h stimulation conditions.

### Correlation network for analysis

Spearman correlation analysis was performed between all pairs of immune features. The correlation network consists of a graph on which each edge represents a significant correlation between the two respective immune features (*p* value < 0.05 after Bonferroni adjustment). The graph layout was calculated using the t-SNE algorithm^49^.

### Elastic Net analysis of mass cytometry data

For a matrix $X$ of all immune features and $Y$ the gravity conditions (µG), a multivariate model was developed to calculate the coefficients $\beta$ for each entity in $X$ to minimize the overall differences from $Y$: $[L(\beta ) = |Y - X\beta |{^2}]$. ${L}_{1}$ and ${L}_{2}$ regularization are applied on the $\beta$ coefficients to reduce the model complexity while allowing inclusion of highly correlated measurements, such that $[L(\beta ) = |Y - X\beta {|^2} + {\lambda_1}|\beta {|_1} + {\lambda_2}|\beta |{_2}]$ where *λ*_1_ and *λ*_2_ are selected by cross-validation.

### Visualizations and data resources

All plots were created in R. Data is available for download at cytobank.org. An R script to reproduce the analysis is publicly available upon request at https://flowrepository.org/experiments/2461.

### Ethical use of de-identified human blood samples

All experimental protocols were approved by the Institutional Review Board (IRB) of Stanford University (IRB-38551) and with the 1964 Helsinki Declaration and its later amendments or comparable ethical standards. The need for informed consent has been waived by the IRB of Stanford University.

## Supplementary Information

Supplementary Figure 1: Gating strategy. Supplementary Figure 2: 18h exposure to sµG did not alter peripheral immune cell frequency. Supplemental Table 1: Mass cytometry antibody panel. Supplemental Table 2: Elastic Net model components differentiating the 1G and simulated microgravity conditions
