# Supplementary material for: Human immune system adaptations to simulated microgravity revealed by single-cell mass cytometry
Source: Sci Rep. 2021 Jun 7;11:11872. doi: 10.1038/s41598-021-90458-2 (PMC8184772; doi:10.1038/s41598-021-90458-2)
Supplement: Supplementary file 1 — Supplementary Figure 1: Gating strategy. Supplementary Figure 2: 18h exposure to sµG did not alter peripheral immune cell frequency. Supplemental Table 1: Mass cytometry antibody panel. Supplemental Table 2: Elastic Net model components differentiating the 1G and simulated microgravity conditions [file 41598_2021_90458_MOESM1_ESM.docx]

**Supplementary Materials: Human immune system adaptations to simulated microgravity revealed by single-cell mass cytometry**

**Authors:** J.M. Spatz,^1‡^ M. Hughes Fulford,^1*‡^ A. Tsai,^2^ D. Gaudilliere,^3^ J. Hedou,^2^ E. Ganio,^2^ M. Angst,^2^ N. Aghaeepour,^2‡^ B. Gaudilliere^2*‡^

Supplementary Figure 1: Gating strategy

Supplementary Figure 2: 18h exposure to sµG did not alter peripheral immune cell frequency.

Supplementary Figure 3: Multivariable modeling of basal immune cell adaptions to sµG.

Supplemental Table 1: Mass cytometry antibody panel

Supplemental Table 2: Elastic Net model components differentiating the 1G and simulated microgravity conditions

Supplemental Table 3. Basal EN model components.

\
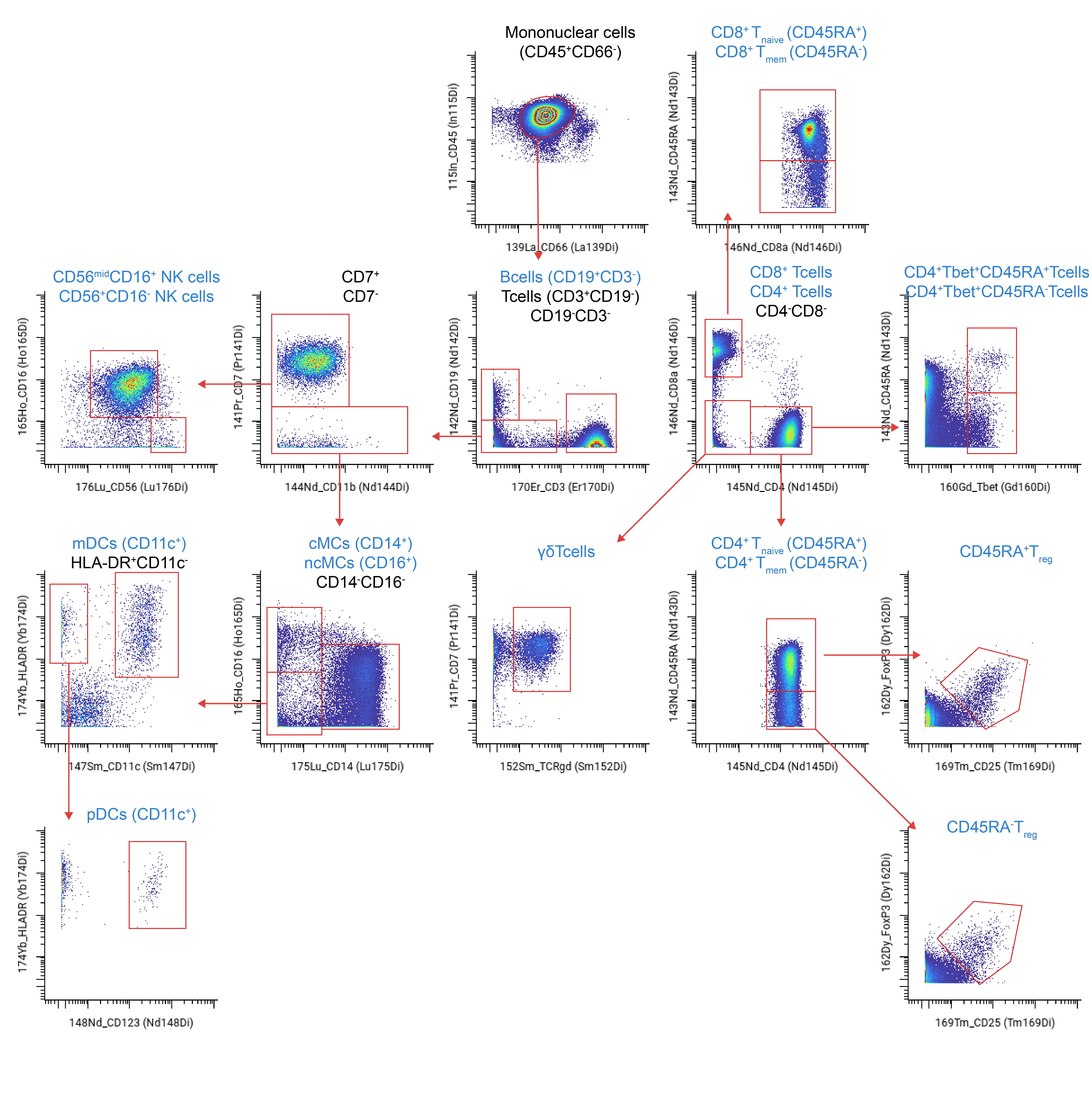


**
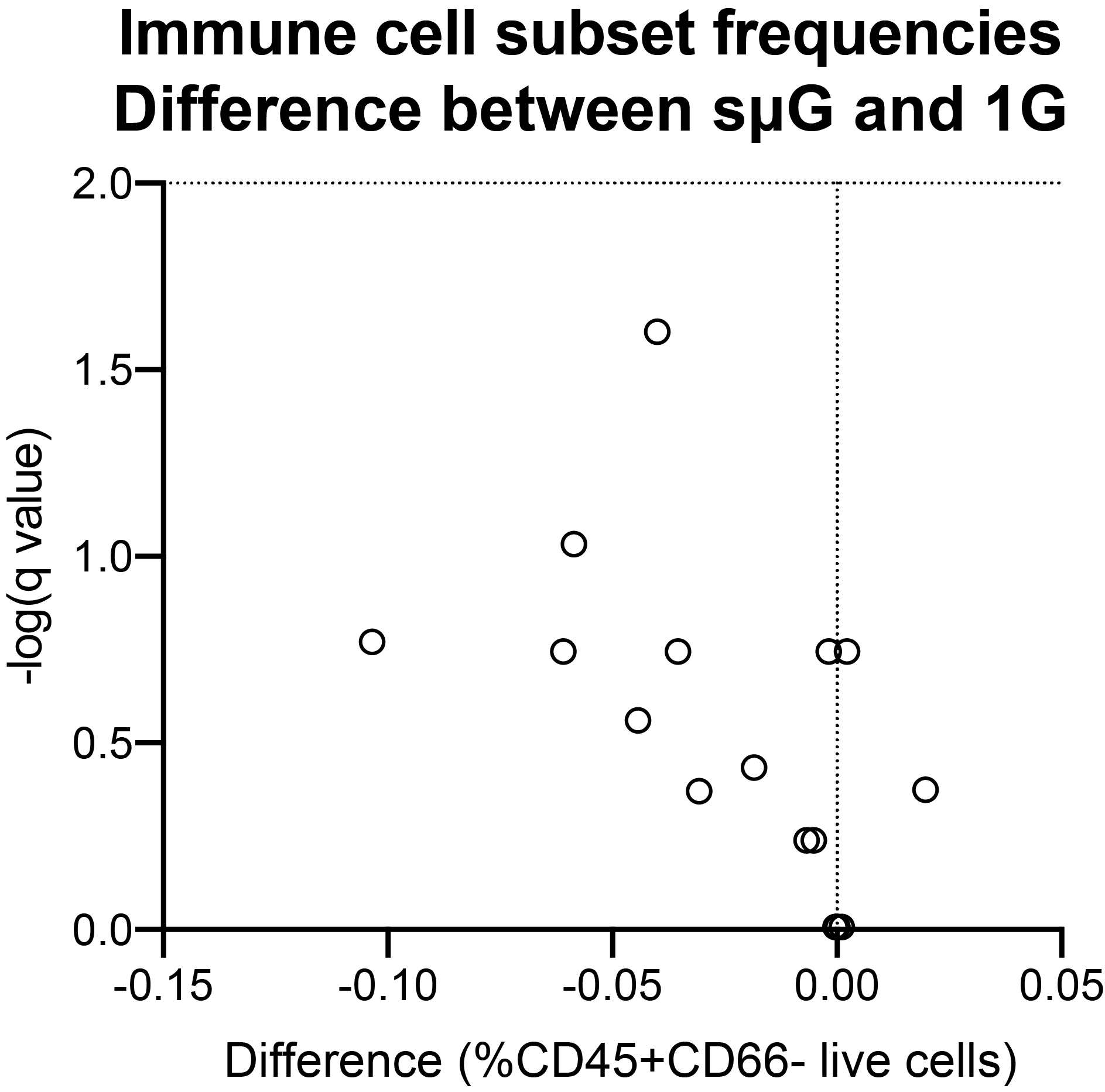
Supplemental Figure 1. Gating Strategy.** Manual gates derived from 2-dimensional scatter plots in Immune Atlas ([https://immuneatlas.org/](https://immuneatlas.org/#/experiments/5b764e34a055f8082609d666/gating)). Cell subsets included in the analysis are highlighted in blue and include (CD4^+^ T, CD4^+^_naïve_T, CD4^+^_mem_T, CD45RA^+^T_reg_, CD45RA^-^T_reg_, TCRγδ T, Tbet^+^CD45RA^+^CD4^+^ T, Tbet^+^CD45RA^-^CD4^+^ T, CD8^+^ T, CD8^+^_naïve_T, CD8^+^_mem_T, CD56midCD16+ Natural Killer (NK), CD56+CD16-NK, classical monocytes (cMC), non-classical monocytes (ncMCs), myeloid (m) and plasmacytoid (p) dendritic (DCs), and B cells

**Supplemental Figure 2. 18h exposure to sµG did not alter peripheral immune cell frequency.** The frequency of the 18 Immune cell subsets included in the analysis were caculated as %CD45+CD66- live cells. The dot plot illustrates -log (q value) comparing the sµG and 1G conditions (18h exposure, unstimulated cells). All frequencies were below the horizontal line at y=2 (FDR q > 0.01, n=8).


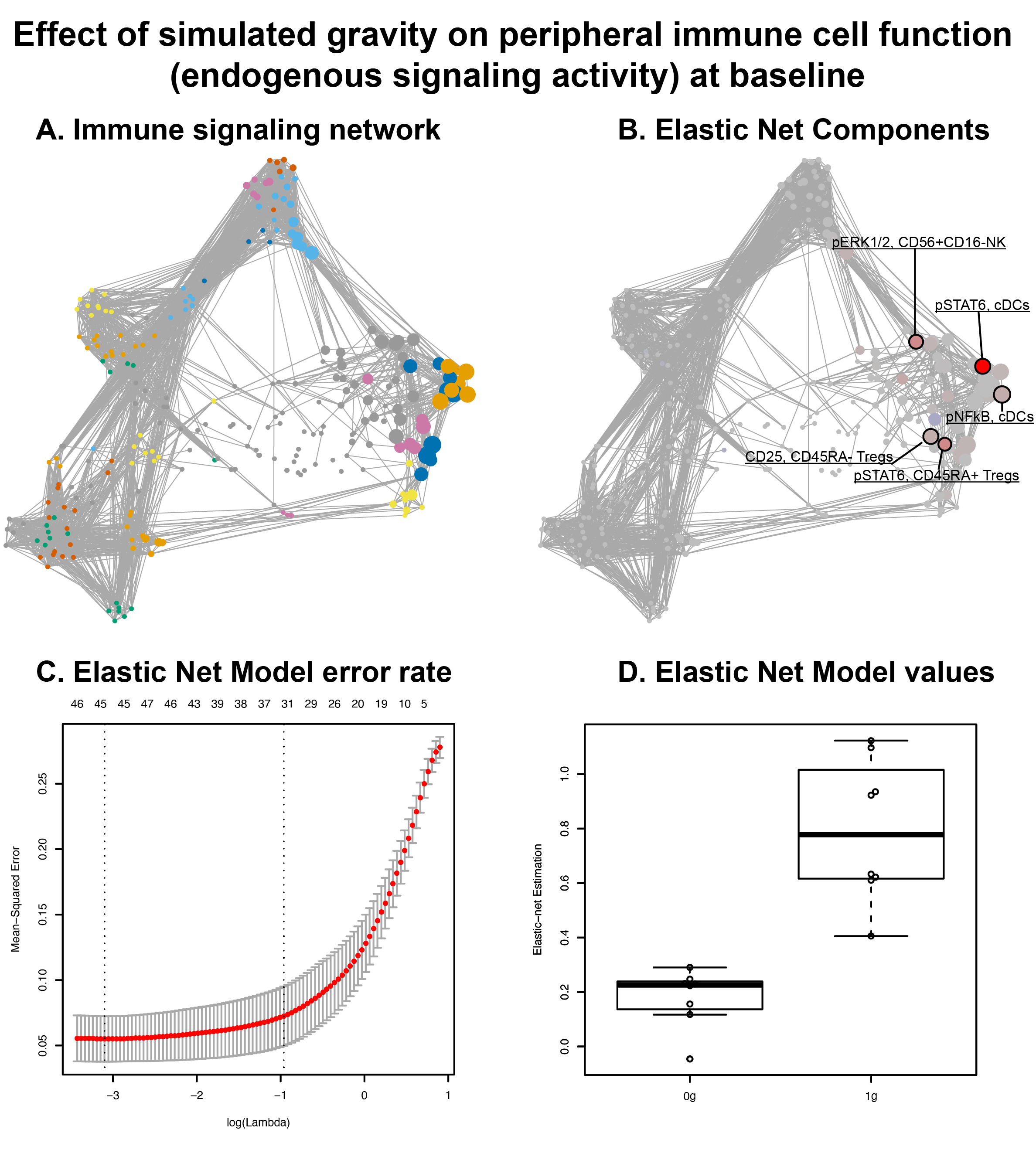


**Supplemental Figure 3: Multivariable modeling of basal immune cell adaptions to sµG. A.** Correlation network depicting single-cell immune responses at baseline. Nodes represent the protein expression or phosphorylation level of one of 14 functional proteomic markers for a given immune cell subset (asinh ratio relative to the 0h timepoint). Edges are proportional to the Spearman correlation between two nodes. The network segregates into communities that were characterized based on the functional attribute that appeared most frequently in each community. **B.** An EN method analysis of immune cell responses that differentiated samples exposed to sµG or 1G. **C.** The graph depicts model error rates after cross-validation across a range of regularization threshold [log(lambda)]. EN models with the lowest error rate (left vertical dotted line) and one standard deviation from the lowest error rate (right vertical line) are indicated. **D.** Output corresponding to the basal EN model one standard deviation from the lowest error rate (p-value=3.7E-4, cross-validation, n=8).

**Supplemental Table 1. Mass cytometry antibody panel.**

| Antibody | Symbol | Atomic Mass | Comment |
| --- | --- | --- | --- |
| Barcode 1 | Pd | 102 | Barcode |
| Barcode 2 | Pd | 104 | Barcode |
| Barcode 3 | Pd | 105 | Barcode |
| Barcode 4 | Pd | 106 | Barcode |
| Barcode 5 | Pd | 108 | Barcode |
| Barcode 6 | Pd | 110 | Barcode |
| CD235ab* | In | 113 | Phenotype |
| CD61* | In | 113 | Phenotype |
| CD45 | In | 115 | Phenotype |
| CD66 | La | 139 | Phenotype |
| CD7 | Pr | 141 | Phenotype |
| CD19 | Nd | 142 | Phenotype |
| CD45RA | Nd | 143 | Phenotype |
| CD11b | Nd | 144 | Phenotype |
| CD4 | Nd | 145 | Phenotype |
| CD8a | Nd | 146 | Phenotype |
| CD11c | Sm | 147 | Phenotype |
| CD123 | Nd | 148 | Phenotype |
| pCREB | Sm | 149 | Function |
| pSTAT5 | Nd | 150 | Function |
| pp38 | Eu | 151 | Function |
| TCRγδ | Sm | 152 | Phenotype |
| pSTAT1 | Eu | 153 | Function |
| pSTAT3 | Sm | 154 | Function |
| pS6 | Gd | 155 | Function |
| CXCR3 | Gd | 156 | Phenotype |
| CD69 | Gd | 157 | Phenotype |
| CD33 | Gd | 158 | Phenotype |
| pMAPKAPK2 | Tb | 159 | Function |
| Tbet | Gd | 160 | Phenotype |
| cPARP | Dy | 161 | Function |
| FoxP3 | Dy | 162 | Phenotype |
| IκB | Dy | 164 | Function |
| CD16 | Ho | 165 | Phenotype |
| pNF-κB | Er | 166 | Function |
| pERK1/2 | Er | 167 | Function |
| pSTAT6 | Er | 168 | Function |
| CD25 | Tm | 169 | Phenotype |
| CD3 | Er | 170 | Phenotype |
| CCR7 | Yb | 171 | Phenotype |
| CD15 | Yb | 172 | Phenotype |
| CCR2 | Yb | 173 | Phenotype |
| HLA-DR | Yb | 174 | Phenotype |
| CD14 | Yb | 175 | Phenotype |
| CD56 | Yb | 176 | Phenotype |
| DNA1 | Ir | 191 | DNA |
| DNA2 | Ir | 192 | DNA |

**Supplemental Table 2. Model components.** Discovery determined using the two-stage linear step-up procedure of Benjamini, Krieger and Yekutieli, with Q = 1% in Prism. Each row was analyzed individually, without assuming a consistent SD. Number of t tests: 252.

| Feature | P value | Mean of uG | Mean of 1G | q value |
| --- | --- | --- | --- | --- |
| CD8_T_cells_CD69 | 0.000001 | 0.936 | 2.86 | 0.000021 |
| CD4__T_cell_naive_CD25 | 0.000001 | -0.0292 | 0.432 | 0.000021 |
| CD56midCD16_NKcells_CD25 | 0.000001 | 0.0115 | 0.22 | 0.000022 |
| Tbet_CD45RA-CD4__T_cells_CD69 | 0.000002 | 2.75 | 4.28 | 0.000032 |
| CD45RA-_Tregs_STAT5 | 0.000004 | 2.41 | 0.369 | 0.00006 |
| CD8__T_cells_naive_STAT1 | 0.000005 | 0.558 | 1.45 | 0.000061 |
| CD4__T_cell_mem_CD69 | 0.000005 | 1.5 | 3.47 | 0.000061 |
| CD8__T_cells_naive_STAT5 | 0.000013 | 0.199 | 1.17 | 0.000146 |
| CD56_CD16-NKcells_CREB | 0.000015 | 0.662 | -0.11 | 0.000153 |
| CD4_T_cells_CD25 | 0.000015 | -0.17 | 0.269 | 0.000153 |
| CD4__T_cell_mem_CD25 | 0.000032 | -0.203 | 0.271 | 0.000309 |
| CD45RA__Tregs_STAT5 | 0.000048 | 2.18 | 0.628 | 0.000437 |
| CD8___T_cells_mem_STAT1 | 0.000076 | 0.114 | 0.575 | 0.000662 |
| CD8__T_cells_naive_STAT3 | 0.000126 | 0.686 | 1.68 | 0.001058 |
| Tbet_CD45RA_CD4__T_cells_CD69 | 0.00014 | 2.25 | 3.72 | 0.001122 |
| CD8_T_cells_STAT1 | 0.000182 | 0.436 | 1.2 | 0.001406 |
| CD4__T_cell_naive_S6 | 0.00019 | 0.193 | 1.03 | 0.001416 |
| pDCs_STAT3 | 0.000273 | 0.259 | 1.85 | 0.001956 |
| ncMCs_ERK | 0.00031 | -0.358 | -1.18 | 0.002147 |
| Tbet_CD45RA_CD4__T_cells_CD25 | 0.000338 | 0.0371 | 0.491 | 0.002259 |
| CD56_CD16-NKcells_STAT3 | 0.000348 | 0.756 | 1.26 | 0.002259 |
| Tbet_CD45RA-CD4__T_cells_STAT1 | 0.000386 | 0.451 | 1.2 | 0.002423 |
| Tbet_CD45RA-CD4__T_cells_pSTAT6 | 0.00041 | 0.304 | 1.32 | 0.002499 |
| CD8___T_cells_mem_STAT3 | 0.000567 | 0.576 | 1.52 | 0.003349 |
| CD8_T_cells_STAT5 | 0.000714 | 0.289 | 1.05 | 0.004101 |
| CD8__T_cells_naive_p38 | 0.000765 | 0.01 | 0.178 | 0.004273 |
| CD8__T_cells_naive_pSTAT6 | 0.000849 | 0.573 | 1.89 | 0.00461 |
| CD56midCD16_NKcells_pSTAT6 | 0.000937 | 0.198 | 0.946 | 0.004787 |
| CD56_CD16-NKcells_pSTAT6 | 0.000952 | 0.506 | 1.51 | 0.004787 |
| CD8_T_cells_STAT3 | 0.000971 | 0.682 | 1.61 | 0.004787 |
| CD56midCD16_NKcells_CD69 | 0.000977 | 0.764 | 1.42 | 0.004787 |
| mDCs_ERK | 0.001 | -0.0489 | -0.499 | 0.004787 |
| Tbet_CD45RA_CD4__T_cells_STAT3 | 0.001147 | 0.49 | 1.29 | 0.005359 |
| Tbet_CD45RA_CD4__T_cells_pSTAT6 | 0.001226 | 0.444 | 1.61 | 0.005601 |
| CD56midCD16_NKcells_STAT1 | 0.001448 | 0.0174 | 0.164 | 0.006466 |
| mDCs_NFkB | 0.001512 | -0.0207 | -0.294 | 0.006605 |
| CD8__T_cells_naive_S6 | 0.00155 | 0.285 | 0.903 | 0.006629 |
| CD56midCD16_NKcells_STAT5 | 0.00165 | 0.268 | 0.86 | 0.00684 |
| CD4__T_cell_naive_STAT5 | 0.001667 | 0.448 | 0.985 | 0.00684 |
| CD8___T_cells_mem_CD69 | 0.001785 | 1.59 | 2.64 | 0.007177 |
| mDCs_cPARP | 0.001893 | 0.0836 | 0.374 | 0.007461 |
| CD45RA-_Tregs_CD25 | 0.001947 | -0.0872 | -0.454 | 0.007527 |
| CD8___T_cells_mem_pSTAT6 | 0.002022 | 0.297 | 1.26 | 0.007668 |
| CD45RA__Tregs_S6 | 0.002198 | 0.28 | 1.03 | 0.008183 |
| CD45RA__Tregs_pSTAT6 | 0.002339 | 0.41 | 1.42 | 0.008548 |
| CD4_T_cells_S6 | 0.002734 | 0.256 | 0.892 | 0.009662 |
| B-cells_S6 | 0.00274 | 1.31 | 0.484 | 0.009662 |
| CD8_T_cells_pSTAT6 | 0.002814 | 0.505 | 1.67 | 0.009751 |
| Tbet_CD45RA-CD4__T_cells_STAT3 | <0.000001 | 0.377 | 1.8 | 0.000003 |
| CD4__T_cell_naive_CD69 | <0.000001 | 0.543 | 3.15 | 0.000004 |
| CD4_T_cells_CD69 | <0.000001 | 1 | 3.31 | 0.00001 |
| CD45RA__Tregs_CD69 | <0.000001 | 0.603 | 3.23 | 0.000003 |
| CD45RA-_Tregs_CD69 | <0.000001 | 1.4 | 3.21 | 0.000008 |
| CD8__T_cells_naive_CD69 | <0.000001 | 0.688 | 2.89 | 0.000011 |
| CD8___T_cells_mem_CD25 | <0.000001 | 0.00815 | 0.41 | 0.000006 |
| CD8__T_cells_naive_CD25 | <0.000001 | 0.00235 | 0.379 | 0.000014 |
| CD8_T_cells_CD25 | <0.000001 | -0.0121 | 0.375 | 0.000002 |
| Tbet_CD45RA-CD4__T_cells_CD25 | <0.000001 | 0.0946 | 0.842 | 0.000019 |

**Supplemental Table 3. Basal EN model components.** Discovery determined using the two-stage linear step-up procedure of Benjamini, Krieger and Yekutieli, with Q = 1% in Prism. Each row was analyzed individually, without assuming a consistent SD. Number of t tests: 252.

| Feature | P value | Mean of ug | Mean of 1g | q value |
| --- | --- | --- | --- | --- |
| CD45RA__Tregs_STAT5 | 0.0001554 | 0.624 | 1.675 | 0.000248 |
| CD45RA-_Tregs_STAT5 | 0.0001554 | 0.5876 | 2.033 | 0.000029 |
| cDCs_MAPKAPK2 | 0.0001554 | 0.5475 | 1.405 | 0.000636 |
| cDCs_p38 | 0.0001554 | 0.2208 | 0.6048 | 0.005287 |
| cDCs_NFkB | 0.0003108 | 0.2065 | 0.5682 | 0.0018 |
| cDCs_IkB | 0.0003108 | 0.2551 | 0.6422 | 0.005992 |
| cDCs_ERK | 0.0003108 | 0.1755 | 0.6468 | 0.010597 |
| cDCs_pSTAT6 | 0.0006216 | 0.06326 | 0.09285 | 0.003478 |
| CD45RA-_Tregs_CD25 | 0.0006216 | 3.26 | 3.729 | 0.002129 |
| B-cells_CD69 | 0.0006216 | 0.7704 | 1.101 | 0.010597 |
| CD56_CD16-NKcells_ERK | 0.0010878 | 0.1235 | 0.2097 | 0.022266 |
| CD45RA__Tregs_S6 | 0.0010878 | 0.4621 | 0.7473 | 0.021735 |
| CD56_CD16-NKcells_STAT5 | 0.0010878 | 0.3699 | 0.6549 | 0.005346 |
| cDCs_STAT5 | 0.0010878 | 0.3646 | 0.6591 | 0.005234 |
| CD45RA__Tregs_CD69 | 0.0010878 | 0.5932 | 0.7557 | 0.013257 |
| CD45RA__Tregs_pSTAT6 | 0.0018648 | 0.4868 | 0.5586 | 0.021735 |
| cMCs_cPARP | 0.0018648 | 0.2144 | 0.1325 | 0.049144 |
| CD45RA-_Tregs_STAT1 | 0.0029526 | 0.2785 | 0.425 | 0.01487 |
| ncMCs_pSTAT6 | 0.00466201 | 0.08068 | 0.1151 | 0.028913 |
| CD45RA__Tregs_STAT3 | 0.00466201 | 0.6975 | 1.225 | 0.022266 |
| CD56_CD16-NKcells_CREB | 0.00466201 | 1.298 | 1.841 | 0.042431 |
| ncMCs_ERK | 0.00466201 | 0.583 | 1.306 | 0.022266 |
| cDCs_CD69 | 0.00699301 | 0.3329 | 0.6042 | 0.054422 |
| CD45RA__Tregs_CREB | 0.00699301 | 1.188 | 1.69 | 0.035115 |
| CD45RA-_Tregs_p38 | 0.01041181 | 0.3847 | 0.5652 | 0.031462 |
| CD56_CD16-NKcells_CD69 | 0.01041181 | 1.403 | 1.781 | 0.054422 |
| cMCs_S6 | 0.01476302 | 1.415 | 1.77 | 0.102331 |
| CD56midCD16_NKcells_NFkB | 0.02066822 | 1.028 | 0.8083 | 0.109205 |
| CD56midCD16_NKcells_IkB | 0.13038073 | 1.713 | 1.492 | 0.197484 |
| B-cells_IkB | 0.13038073 | 1.834 | 1.6 | 0.208385 |
| CD56midCD16_NKcells_STAT1 | 0.38228438 | 0.205 | 0.1903 | 0.554006 |
